# The Length of Incision in Unicondylar Knee Arthroplasty Is More Affected by the Patient’s Height Than Their Weight

**DOI:** 10.7759/cureus.44444

**Published:** 2023-08-31

**Authors:** Selçuk Yılmaz, Nihat Demirhan Demirkıran, Sabit Numan Kuyubaşı, Suleyman Kozlu, Mehmet Kurt, Alper Akkurt, S. Kaan Öner

**Affiliations:** 1 Orthopedics and Traumatology, Kütahya Health Sciences University, Kütahya, TUR; 2 Orthopedics and Traumatology, Private Cankaya Hospital, Ankara, TUR

**Keywords:** incision technique, arthroplasty incision, incision length, incision, unicondylar knee prosthesis

## Abstract

Objective

Obesity leads to osteoarthritis due to increased loading forces on joint cartilage and inflammatory agents released from adipose tissue. In patients with a high body mass index (BMI), during hip and total knee arthroplasty, surgical technical challenges such as longer incisions and wider exposure are encountered, resulting in increased postoperative complications (wound healing problems and infection, venous thromboembolism (VTE)- pulmonary embolism (PE), dislocation, early implant failure) and ultimately decreased patient satisfaction and implant survival. This study investigates whether BMI, height, weight, and patient age are associated with longer incisions in patients undergoing unicondylar knee prosthesis (UKP) placement.

Method

Between January 2017 and December 2018, 30 patients (29 females and 1 male) who underwent UKP surgery due to medial gonarthrosis were included in the study. The UKP used in the procedures was the Oxford Knee Phase III by Biomet Ltd., UK. The study comprised 43 knees, 13 being bilateral cases, 8 on the right, and 9 on the left. Data regarding the patient's height, weight, BMI, age, and the operated side were collected and compiled. The relationships between these variables and the surgical incision length were statistically analyzed.

Results

The average age of the patients was 66.3 years, with an average weight and height of 77.6 kg (ranging from 62 to 98 kg) and 167 cm (ranging from 150 to 184 cm), respectively. The lengths of the surgical incisions ranged from 70 mm to 160 mm, with an average length of 124.5 mm. When comparing the incision lengths between the right and left sides, it was observed that the incisions on the left side were longer. The average incision length on the right side was 122.09 mm, while on the left, it was 126.86 mm. Moreover, in the 13 patients who underwent bilateral surgery, this difference in incision length was even more pronounced. The average incision length on the right side was 117.15 mm, whereas on the left, it was 124.23 mm. Bivariate correlation analyses were performed to examine the relationship between the length of the incision and BMI and age. However, no significant relationship was found between the incision length and BMI or age. On the other hand, there was a correlation between the patient's weight values and the incision length (p < 0.05, correlation 0.335). Furthermore, a higher correlation was observed between the patient's height and the incision length (p < 0.01, correlation 0.595).

Conclusion

The latest advances in surgical techniques and instrumentation have enabled surgeons to perform the procedure using a reliable mini-incision approach. Mid-term evaluation of UKP with mini-incision shows faster recovery and lower morbidity. The findings show that in UKP, the length of the surgical incision is more strongly related to the patient's height than their weight.

## Introduction

Osteoarthritis (OA) is a clinical syndrome of joint pain associated with varying degrees of functional limitation and poor quality of life. Pathologically, it is characterized by localized cartilage loss, adjacent bone remodeling, and consequent inflammation [1]. Studies have shown an association between generally high BMI, obesity, and the prevalence and incidence of knee OA. Obesity has been suggested as the main modifiable risk factor in osteoarthritis [2,3]. Operating on obese patients is technically difficult, and complications may occur during implant placement due to difficulties and inability to obtain adequate surgical clearance. In all obese TKA patients, a midline incision is appropriate, usually longer than a standard incision, and performed with the knee in flexion. Extending the subfascial dissection on the lateral side of the knee is helpful to create a pocket into which the extensor mechanism can be everted. This pocket avoids extra tension on the extensor mechanism to prevent patellar tendon avulsion, allows control of the patella within the fat pocket, and prevents doubling over the skin with attendant vascular compromise [4,5]. Minimally invasive techniques have been developed to reduce surgical trauma associated with knee arthroplasty. Potential benefits of minimal incision surgery include less surgical dissection, less blood loss and pain, faster return to social life, a smaller scar, and lower costs [6]. The main advantage of the minimally invasive procedure is that the patient recovers faster and returns to social life [7].

In our current study, the focal point is investigating the potential correlations between BMI, height, weight, patient age, and the length of incisions in individuals who have undergone unicondylar knee prosthesis (UKP) procedures. While the clinical relevance of BMI and its relationship with surgical outcomes has been explored extensively, this study aims to delve into the nuanced associations between patient characteristics and incision length, specifically within the context of UKP surgeries. By shedding light on these relationships, we strive to glean insights that could further refine surgical techniques and postoperative care strategies, ultimately enhancing the overall experience and outcomes for patients undergoing UKP procedures.

## Materials and methods

**Figure 1:**
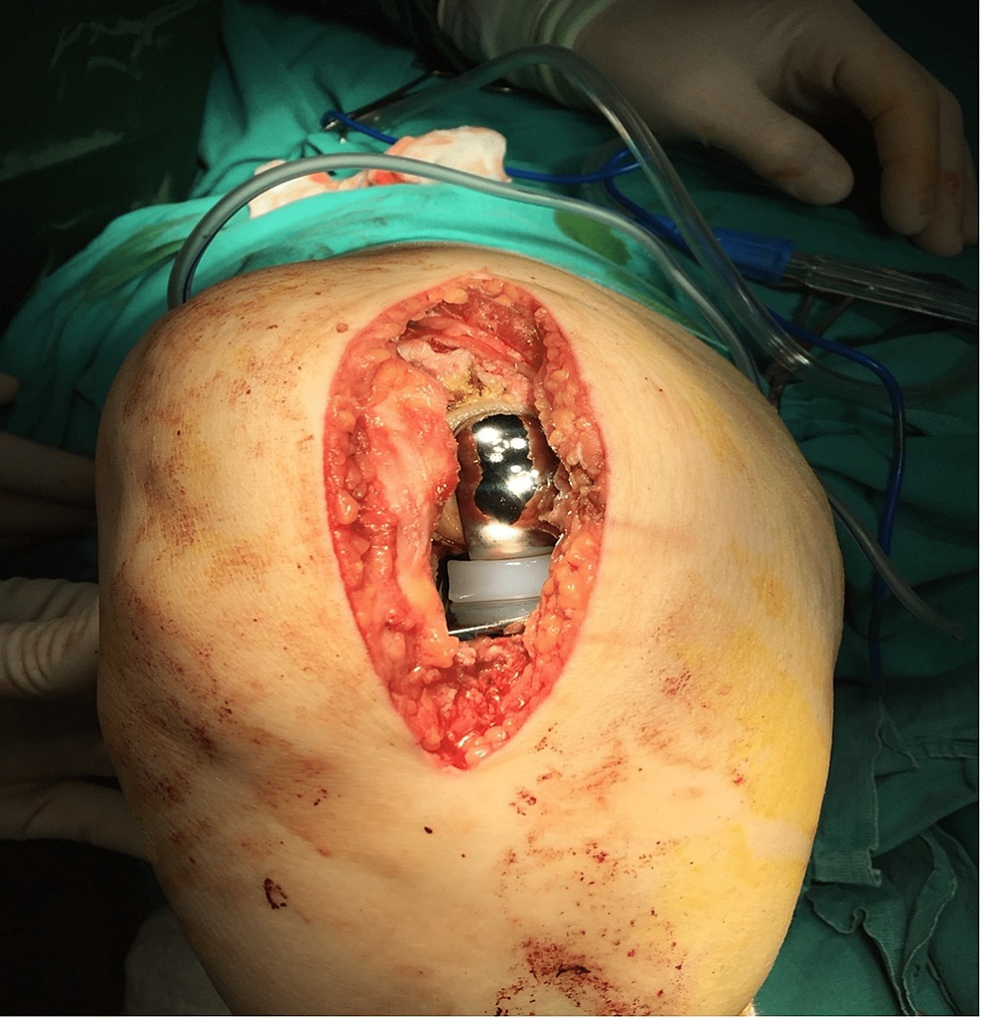
Appearance of skin incision during flexion

Between January 2017 and December 2018, 43 knees (13 bilateral, 8 right, 9 left) of 30 patients (29 female, 1 male) who underwent UKP (Oxford Knee Phase III, Biomet Ltd., UK) due to medial gonarthrosis were included in the study. Our exclusion criteria are revision cases, patients who have undergone previous knee surgery, and patients with patellofemoral and lateral arthrosis. We added it to the relevant section. The patient's height, weight, BMI, age, and operated side data were compiled, and their relationship with the surgical incision length was statistically analyzed. The procedure was performed under spinal anesthesia and on a routine operating table, with the knee flexed 90° for skin incision, the thigh tourniquet inflated, and the foot resting on the table. A paramedial incision was routinely made from the patella's superomedial edge to the tibial tubercle's medial border. After the implantation of the prosthesis, at the end of the procedure, the incision size was measured with the knee in 90-degree flexion and the tourniquet inflated, with the foot resting on the table (Figure 1)-a single surgeon operated on the patient. After the tourniquet is released before closing for adequate hemostasis, bleeding is controlled, and a drain is placed. Appropriate antibiotic prophylaxis was administered preoperatively in each patient. Our study used the Pearson correlation coefficient method to evaluate the relationship between variables. In addition, to determine the statistical significance of each relationship in our study, Sig. We have used the value (2-tailed). Pearson correlation coefficients obtained in our study, Sig. We have detailed the relationship between variables using values and sample sizes. This way, we could better understand the relationships between incision length and height, weight, BMI, and age.

## Results

The mean age of the patients was 66.3, mean weight and height were 77.6 (62-98) kg and 167 (150-184) cm, respectively. The length of the incisions varied between 70mm and 160mm (mean 124.5). Comparing the incision lengths of 22 right and 21 left sides, the left sides were longer (mid right: 122.09; left: 126.86 mm); It was observed that this difference was even more pronounced in the knees of 13 bilaterally operated patients (mid right: 117.15; left: 124.23 mm). In bilateral correlation analyses, no significant correlation was observed between incision length, BMI, and age. A correlation was observed between the weight values of the patients and the incision length. (p<0.05, correlation 0.335) The correlation between the height of the patients and the incision length was found to be higher. (p<0.01, correlation 0.595) (Table 1).

**Table 1:** Statistical data of our study. Sig.= Significance; BMI= Body Mass Index. **: Correlation is significant at the 0.01 level (2-tailed). *: Correlation is significant at the 0.05 level (2-tailed).

	Incision	Height	Weight	BMI	Age	
Incision	Pearson Correlation	1	0.595**	0.335*	-0.128	-0.107
Sig. (2-tailed)		0	0.028	0.412	0.496	
N	43	43	43	43	43	
Height	Pearson Correlation	0.595**	1	0.243	-0.475**	-0.127
Sig. (2-tailed)	0		0.116	0.001	0.416	
N	43	43	43	43	43	
Weight	Pearson Correlation	0.335*	0.243	1	0.734**	0.007
Sig. (2-tailed)	0.028	0.116		0	0.966	
N	43	43	43	43	43	
BMI	Pearson Correlation	-0.128	-0.475**	0.734**	1	0.108
Sig. (2-tailed)	0.412	0.001	0		0.49	
N	43	43	43	43	43	
Age	Pearson Correlation	-0.107	-0.127	0.007	0.108	1
Sig. (2-tailed)	0.496	0.416	0.966	0.49		
N	43	43	43	43	43	

## Discussion

Few reports are available during interim follow-up for the evaluation of UKP via mini-incision. Argenson et al. In their study with minimally invasive UKP, they reported that mass and soft tissue flexibility would affect the incision length; they tried a minimally invasive surgical incision technique in their study and reported that the average incision length varied between 6-8 cm. They predicted that this length difference varies according to the patient's obesity and tissue elasticity [8]. In our study, the mean incision length was 12.4 cm. Fuchs et al. Using electromyography, gait analysis, and quality of life scores in 29 patients who were operated on with a mini-incision and underwent unicondylar knee arthroplasty, they reported that UKP performed with a mini-incision had a higher quality of life than total knee arthroplasty and had functional parameters comparable to healthy individuals of the same age [8]. Clinical and functional results in all studies report that patients tend to recover faster after UKP through mini-incision [8-10]. However, Hamilton et al. compared 221 cases of UKP performed with a mini-incision to 514 cases performed with an open technique, reporting aseptic loosening of 1% in the open technique group and 3.7% in the mini-incision group [11]. Although a minimal incision yields better functional outcomes, having a sufficient incision area for proper joint alignment is also crucial in arthroplasty, as revisions are more likely to occur in the early stages due to alignment errors. However, the widely accepted threshold of 10-year implant survival for joint reconstruction procedures has not yet been evaluated in any study.

Non-obese individuals tend to have better long-term functional scores, making weight management beneficial for patients requiring UKP. Excessive weight or high BMI (BMI ≥ 30 kg/m^2^) will increase component wear, thus reducing the prosthesis survival rate [12,13]. Additionally, obesity's adverse effects on UKP include excessive incision tension, delayed healing, higher infection rates, and lower activity levels during later rehabilitation training [14]. A study comparing mini-incision to standard incision in terms of alignment reported similar results for both incision types after surgical intervention [15]. We believe that minimal incisions in UKP would yield satisfactory results in terms of both alignment and functional outcomes.

In their study, Izaka reported that patients with lower BMI have lower skin elasticity [16]. Our study also observed an increase in skin incision length with an increase in BMI. However, we found that the increase in incision length was even more significant with an increase in height. McPherson et al. conducted a study on total knee arthroplasty and suggested that incision length is mostly related to the width of the knee bone. They measured incision length in extension and proposed that an incision length approximately 1.6 times the width of the distal femur could be a sufficient criterion for a minimally invasive incision [17]. However, our study found a stronger correlation between incision length and the patient's height than their weight. Our study found a strong correlation between height and incision length. Complications related to wound problems, infection, and bleeding increase with incision size. For this reason, preoperative measures should be taken against these complications in tall patients [5,11,14]. It should be noted that stronger statistical results can be obtained in larger case samples; multicenter studies with higher numbers of cases are important in this regard.

Limitation

Our study was limited in a number of cases. Inter-case evaluation could also be made regarding long-term clinical outcomes and joint range of motion. Single surgeons had no bias; we thought this would be more objective. The relationship between the incision length, the surgeon's dominant hand use, and the patient's side should not be overlooked.

## Conclusions

The latest advances in surgical techniques and instrumentation have enabled surgeons to perform the procedure using a reliable mini-incision approach. Mid-term evaluation of UKP with mini-incision shows faster recovery and lower morbidity. In UKP, we believe incision length is more strongly related to the patient's height than weight. We think the longer incision length in the left knee may be due to the difficulty during the incision on the left side because we use the right hand dominantly. New comprehensive studies will guide us on the unicondylar knee prosthesis surgical technique.
